# Pharmacological interventions in the treatment of the acute effects of cannabis: a systematic review of literature

**DOI:** 10.1186/1477-7517-9-7

**Published:** 2012-01-25

**Authors:** José AS Crippa, Guilherme N Derenusson, Marcos HN Chagas, Zerrin Atakan, Rocio Martín-Santos, Antonio W Zuardi, Jaime EC Hallak

**Affiliations:** 1 Department of Neurosciences and Behavior, Ribeirão Preto Medical School, University of São Paulo and INCT Translational Medicine (CNPq), Brazil; 2 Section of Neuroimaging, Box PO67, Division of Psychological Medicine & Psychiatry, Institute of Psychiatry, King's College London, De Crespigny Park, London, SE5 8AF, UK; 3 Department of Psychiatry, Institute of Neurosciences, Hospital Clínic, IDIBAPS, CIBERSAM, Barcelona, Spain and INCT Translational Medicine (CNPq), Brazil

**Keywords:** cannabis, intoxication, acute effects, psychosis, anxiety, pharmacological treatment

## Abstract

**Background:**

Cannabis intoxication is related to a number of physical and mental health risks with ensuing social costs. However, little attention has been given to the investigation of possible pharmacological interactions in this condition.

**Objective:**

To review the available scientific literature concerning pharmacological interventions for the treatment of the acute effects of cannabis.

**Methods:**

A search was performed on the *Pubmed*, *Lilacs*, and *Scielo *online databases by combining the terms *cannabis, intoxication, psychosis, anxiety*, and *treatment*. The articles selected from this search had their reference lists checked for additional publications related to the topic of the review.

**Results:**

The reviewed articles consisted of case reports and controlled clinical trials and are presented according to interventions targeting the physiological, psychiatric, and cognitive symptoms provoked by cannabis. The pharmacological interventions reported in these studies include: beta-blockers, antiarrhythmic agents, antagonists of CB-1 and GABA-benzodiazepine receptors, antipsychotics, and cannabidiol.

**Conclusion:**

Although scarce, the evidence on pharmacological interventions for the management of cannabis intoxication suggests that propanolol and rimonabant are the most effective compounds currently available to treat the physiological and subjective effects of the drug. Further studies are necessary to establish the real effectiveness of these two medications, as well as the effectiveness of other candidate compounds to counteract the effects of cannabis intoxication, such as cannabidiol and flumazenil.

## Introduction

*Cannabis sativa *has Δ^9^-tetrahydrocannabinol (Δ^9^-THC), isolated in the 1960s [1], as its main psychoactive compound. The concentration of Δ^9^-THC in the different presentations of cannabis (marijuana, hashish, skunk) is proportional to the intensity of its toxic effects. In cannabis, considered to be the most consumed illicit substance in the world [2], increased concentrations of Δ^9^-THC have been reported in recent years [3-5].

At the same time, there has been an increase in the number of patients reporting to specialized services with complaints related to the use of cannabis. A survey in the USA revealed that between 1992 and 1998 the demand for treatment at specialized services has doubled, with the percentage of admissions for the treatment of cannabis-related disorders (23%) approaching the admission rates relative to cocaine (27%) and heroine (23%), considering all drug-related hospitalizations [6].

The use of cannabis has been associated with several psychological, behavioral, and social problems [2]. Besides the chronic effects of the continued use of cannabis, such as dependence, abstinence, varying degrees of cognitive impairment, and increased risk of respiratory disorders, its acute effects have also been related to significant physical and mental health problems [7,8], and an increasing number of emergency admissions has been linked to cannabis use [9,10].

The intoxication by cannabis is associated with subjective symptoms of euphoria, perceptual distortion, continuous giggling, sedation, lethargy, impaired perception of time, difficulties in the performance of complex mental processes, impaired judgment and social withdrawal [11]. In addition, physical signs of conjunctival hyperemia, increased appetite, dry mouth, and tachycardia can develop in the period of approximately two hours after the use of the substance [11], corresponding to the plasmatic peak of Δ^9^-THC.

In general, the acute toxicity of cannabinoids is considered to be low. Nevertheless, there are reports of death by brain infarction - especially among teenagers - following the acute use of marijuana [12,13] as well as of cases of patients with severe sequelae resulting from this complication [14]. Similarly, there are reports of coma in children induced by the accidental intake of cannabis [15], in addition to cases of cardiac arrhythmia [16-20], acute myocardial infarction [21], and transitory ischemic attacks [14]. Factors such as increased heart effort, elevated levels of catecholamine and carboxyhemoglobin in the blood, as well as the occurrence of postural hypotension are among the most commonly reported factors of cardiovascular disease associated with intoxication by cannabis [22].

It is known that cannabis intoxication leads to impaired motor ability, attention, and short-term memory [8,23]. In accordance with this, many studies have found a higher prevalence of cannabis use among drivers involved in accidents than in the general population [8].

Currently, there is consistent evidence that people who use cannabis on a regular basis have a higher proportion of acute psychiatric disorders, aggravated by other factors such as personality traits, pre-existing vulnerability, and substance use at an early age [24]. First-time use and the dose of cannabis are among the main factors related to this occurrence [23].

Panic and anxiety attacks are among the most commonly reported psychiatric symptoms related to cannabis intoxication and are often responsible for the discontinuation of the use of the substance [23]. Acute psychotic episodes related to cannabis intoxication are described in terms of confusion, disorientation, amnesia, depersonalization, delusions, hallucinations, paranoid ideation, psychomotor agitation, labile affect, and hostility [25]. These symptoms are usually gone after a maximum of one week abstinence [26]. In some cases, psychotic episodes secondary to the use of cannabis can persist for a substantial period of time after the acute intoxication and may have some of the features of acute schizophreniform disorders [24,27,28]. Further evidence is provided by a systematic review on longitudinal and population-based studies, which showed that cannabis use significantly increases the risk of developing psychotic illnesses in a dose-dependent manner [29].

There is strong evidence that cannabis use can have major detrimental effects on the course of the illness when patients with a pre-existing psychotic condition continue to use the drug [30]. In addition to worsening the outcome and exacerbating the symptoms, cannabis use by people with psychosis can lead to sudden behavioral disturbances such as increased proneness to violence, criminal activity, suspiciousness, and hallucinations [31].

Lately, attempts have been made to better understand the neurobiological mechanisms underlying cannabis-related disorders and the functioning of the endogenous cannabinoid system. There is increasing interest for the development of medications capable to reduce the morbidity of these disorders. Because marijuana intoxication is a major public health problem with a growing demand for assistance at emergency departments, the study of possible pharmacological interventions that might help in the management of the acute effects of cannabis use is of great clinical and social relevance.

## Method

This study is a bibliographic review on pharmacological interventions for the treatment of the acute effects of cannabis. The search for articles was performed on the *PubMed*, *LILACS*, and *SciELO *online databases using the search terms *cannabis, intoxication, psychosis, anxiety*, and *treatment*, and yielded a total of 174 matches. Additionally, an active search was conducted for related articles in the reference lists of the selected publications, which included case reports, controlled clinical trials, clinical trials of psychiatric and non-psychiatric medications, trials that promoted symptom reduction (physical, psychological or cognitive), and investigations related to the period of cannabis intoxication. Inclusion criteria were articles published in English, Spanish or Portuguese, no time limit up to October of 2011. Only those studies involving human samples were included. Taken together, the database and the hand search provided ten articles considered relevant for this review.

The results were subdivided according to interventions targetting the physiological and psychological acute effects of cannabis.

## Results

The analysis of research reports of pharmacological interventions against cannabis-related problems showed that only a few studies have been carried out on this subject. Ten studies that fulfilled the inclusion criteria consisted of case reports and controlled clinical trials.

### Physiological effects

Cardiovascular effects, such as palpitations, are among the best known clinical findings in cannabis intoxication. These symptoms have been suggested to occur as a result of the response induced by catecholamines, released by the action of the drug in the peripheral autonomic system [32]. The use of propranolol in the control of these effects, proposed by Beaconsfield et al. (1972) [33], was assessed in a placebo-controlled trial. Sulkowski et al. (1977) [34] observed that the use of this medication one hour prior to the use of marijuana significantly attenuated the occurrence of tachycardia, as well as the increase in blood pressure and conjunctival hyperemia in six healthy experienced marijuana smokers.

In two studies conducted by Huestis et al. (2001; 2007) [35,36], the compound rimonabant, previously known as SR141716, was also associated with reduced cannabis-induced tachycardia. This drug is an antagonist of CB-1 cannabinoid receptors and presented its effects at different doses (40 mg and 90 mg) administered two hours before the use of marijuana.

The tachycardia reduction observed with the use of propranolol and rimonabant was independent from pharmacokinetic interactions, which suggests a specific action of these compounds in the blockade of the activation of the sympathetic autonomic system related to the use of cannabis [20]. Although the two drugs were administered as a pretreatment in those studies, it is possible that they could be used to control tachycardia and high blood pressure in cannabis intoxication. Other forms of cardiac arrhythmia have been associated with the use of cannabis. The occurrence of arrhythmias such as atrial fibrillation seems to be associated with effects resulting from the parasympathetic stimulation induced by cannabis and observed with the use of higher doses [20]. In case reports describing the occurrence of atrial fibrillation associated with the use of cannabis, antiarrythmic agents like flecainide [20], propafenone [18], and digoxin [16] were successfully employed in the normalization of the cardiac rhythm. According to Fisher et al. (2005) [20], the use of antiarrythmic agents would be efficient in patients with no structural cardiac alterations.

The occurrence of comatose states due to cannabis intoxication is very rare and, when it happens, it usually is a result of accidental ingestion of the drug by children. Flumazenil, an antagonist of the GABA-benzodiazepine receptor complex, is generally indicated for the treatment of benzodiazepine intoxication and as a therapeutic test in coma states of unknown origin. Rubio et al. (1993) [16] described two cases of children admitted at an emergency department in a comatose state with a clinical history suggestive of intoxication and positive laboratory tests for cannabis. Both patients had a total recovery of the level of conscience after the use of flumazenil.

Cannabis intoxication rarely leads to acute respiratory deficiencies. Such deficiencies are more likely to develop in cases of pre-existing pulmonary pathology or polysubstance use. The appropriate therapeutic approach in these cases requires aggressive measures that include ventilatory support and the specific management of alterations associated with metabolic acidosis, infections, bronchospasm, and agitation. The occurrence of isolated respiratory insufficiency caused by depression of the central nervous system induced by cannabis is associated with a better prognosis [37].

The cases of apnea and depression of the central nervous system are potentially fatal and require ventilatory assistance. Flumazenil was successfully used in the cases reported by Rubio et al. (1993) [16], which support its therapeutic use in cases of cannabis-induced coma. This is consistent with other reports of reversal of the acute brain effects of the drug in different conditions, such as hepatic encephalopathy and intoxication by alcohol, carbamazepine, chloral hydrate, isoflurane, and antihistamines [38-40].

### Psychiatric effects

The literature on the pharmacological management of psychotic, anxious, and affective disorders related to cannabis intoxication is limited. There is paucity of substance specific studies, since most are 'substance use' research without specifying the individual substance, such as cannabis.

In the analysis of the pharmacological management of acute psychotic states induced by cannabis, Berk et al. (1999) [41] compared the effects of olanzapine and haloperidol. In this study, 30 patients who fulfilled the DSM-IV criteria for cannabis-induced psychotic disorder were randomly assigned to two groups divided according to medication (10 mg/day each) administered for four weeks. Both patient groups presented symptom reduction according to the psychiatric scales used (BPRS, CGI severity and improvement), with no statistical differences between them. The use of haloperidol, as expected, was associated with a greater occurrence of extrapyramidal side-effects and the consequent use of antiparkinsonian medication. According to some authors, the use of typical antipsychotics could intensify the use of substances, due mainly to the unpleasant side-effects of this type of medication and competitive metabolism with such substances [42]. Along these lines, increasing evidence supports the possible beneficial properties of atypical neuroleptics in schizophrenia associated with substance abuse disorders [43]. Likewise, as anticholinergic effects are among the symptoms of cannabis intoxication, the use of antipsychotics without such side-effects seems to be more appropriate.

Other pharmacological compounds have been tested in the management of the acute subjective states induced by cannabis. The use of rimonabant 90 mg/day administered two hours before the use of marijuana cigarettes promoted an average reduction of 75% in the signs and symptoms of cannabis intoxication measured one hour after use [36]. Subsequently, the same group tested rimonabant in two distinct protocols, for the same purpose: daily use for 15 days in the dose of 40 mg, and a single dose of 90 mg. The daily administration of the lower dose of rimonabant (40 mg) attenuated the acute subjective effects after 8 but not 15 days of use [35]. These findings concerning the use of rimonabant are relevant as they underscore the role of CB-1 receptors in the mediation of cannabis-induced psychological effects, consistent with previous reports from animal studies. Nonetheless, the lack of clinical trials with the acute use of rimonabant and its important side-effect of inducing depressive episodes [44] limit its employment in this context.

The use of propranolol in the dose of 120 mg led to the reduction of the subjective intoxication effects when administered one hour after the use of marijuana cigarettes and lends further support to the possibility - poorly investigated to date - that the action of THC on the brain could be partially mediated by beta-receptors.

Cannabidiol (CBD), a constituent of cannabis devoid of the typical psychological effects induced by other compounds of the plant, significantly attenuated the anxious and psychotic symptoms induced by Δ^9^-THC (0, 5 mg/kg) in healthy volunteers when simultaneously administered in the dose of 1 mg/kg [45]. The reduction in the anxious and psychotic symptoms induced by THC reported in this study opened novel perspectives for its use in the management of psychotic and anxious states, as attested by later studies performed by our group [46-52]. However, the use of this cannabinoid in the clinical practice is still in the experimental phase and requires further investigation [53].

### Cognitive effects

Only one study assessed the efficacy of pharmacological interventions on the attenuation of the cognitive impairment related to cannabis intoxication. Sulkowski et al. (1977) [34], studying the effects of propranolol 120 mg administered one hour after the use of a marijuana cigarette with a controlled quantity of THC, reported no changes in the performance of six volunteers submitted to psychomotricity, attention, memory, and learning tests. This finding, added to the impact of the laboral and educational impairments associated with the use of cannabis, highlights the importance of the search for novel pharmacological interventions aimed at attenuating the cognitive impairment associated with intoxication by cannabis.

## Conclusions

No publications were found related to the treatment of panic attacks and manic and depressive syndromes specifically associated with cannabis intoxication. We predict that in emergency settings medications with acute therapeutic effects for these conditions are used. Thus benzodiazepines, such as lorazepam and alprazolam, recommended for the acute management of panic disorder [54] can be used, but this requires research evidence for further confirmation. Similarly, manic and depressive syndromes, during the intoxication, can be managed by means of medications (benzodiazepines and antipsychotics) that attenuate important acute complaints like insomnia, anxiety, psychomotor agitation, and suicidal ideation. The use of antidepressants and mood stabilizers would be indicated only in the persistence of these symptoms, with duration beyond the intoxication period.

Cannabis intoxication is associated with significant physical and mental health impairment with resulting social costs; nonetheless, relatively few studies to date were aimed at investigating the possible pharmacological interventions in this condition [55]. However scarce, the available evidence suggests that propranolol and rimonabant are valuable tools in the therapeutic arsenal for the management of the physiological (especially cardiovascular) and subjective intoxication effects of cannabis. Flumazenil and cannabidiol were also found to counteract comatose and anxious and psychotic states, respectively, although evidence in this regard still lacks strength.

Our systematic searches revealed that articles on intoxication by cannabis are scarce and report results based on small samples. Future studies evaluating pharmacological interventions directed at the attenuation of the several acute effects induced by cannabis and at promoting greater knowledge regarding its actions are urgently necessary and opportune in the face of the increasing challenge posed by such an important public health problem.

## Competing interests

The authors declare that they have no competing interests.

## Authors' contributions

JASC coordinated the study and drafted the manuscript. GND and JECH participated in the conception, design of the study and helped to draft manuscript. MHNC, ZA, AWZ and RMS contributed content knowledge and made contribuitions to the written manuscript. All authors read and approved the final manuscript.
